# Detection of Cytosolic *Shigella flexneri* via a C-Terminal Triple-Arginine Motif of GBP1 Inhibits Actin-Based Motility

**DOI:** 10.1128/mBio.01979-17

**Published:** 2017-12-12

**Authors:** Anthony S. Piro, Dulcemaria Hernandez, Sarah Luoma, Eric M. Feeley, Ryan Finethy, Azeb Yirga, Eva M. Frickel, Cammie F. Lesser, Jörn Coers

**Affiliations:** a Department of Molecular Genetics and Microbiology, Duke University Medical Center, Durham, North Carolina, USA; b The Francis Crick Institute, London, United Kingdom; c Department of Medicine, Division of Infectious Diseases, Massachusetts General Hospital, Cambridge, Massachusetts, USA; d Department of Microbiology and Immunobiology, Harvard Medical School, Boston, Massachusetts, USA; e Department of Immunology, Duke University Medical Center, Durham, North Carolina, USA; UC Berkeley

**Keywords:** *Burkholderia*, E3 ligase, IpaH9.8, LPS, O antigen, *Shigella*, actin, dynamin, Gram-negative bacteria, guanylate binding protein, interferons, ubiquitination

## Abstract

Dynamin-like guanylate binding proteins (GBPs) are gamma interferon (IFN-γ)-inducible host defense proteins that can associate with cytosol-invading bacterial pathogens. Mouse GBPs promote the lytic destruction of targeted bacteria in the host cell cytosol, but the antimicrobial function of human GBPs and the mechanism by which these proteins associate with cytosolic bacteria are poorly understood. Here, we demonstrate that human GBP1 is unique among the seven human GBP paralogs in its ability to associate with at least two cytosolic Gram-negative bacteria, *Burkholderia thailandensis* and *Shigella flexneri*. Rough lipopolysaccharide (LPS) mutants of *S. flexneri* colocalize with GBP1 less frequently than wild-type *S. flexneri* does, suggesting that host recognition of O antigen promotes GBP1 targeting to Gram-negative bacteria. The targeting of GBP1 to cytosolic bacteria, via a unique triple-arginine motif present in its C terminus, promotes the corecruitment of four additional GBP paralogs (GBP2, GBP3, GBP4, and GBP6). GBP1-decorated *Shigella* organisms replicate but fail to form actin tails, leading to their intracellular aggregation. Consequentially, the wild type but not the triple-arginine GBP1 mutant restricts *S. flexneri* cell-to-cell spread. Furthermore, human-adapted *S. flexneri*, through the action of one its secreted effectors, IpaH9.8, is more resistant to GBP1 targeting than the non-human-adapted bacillus *B. thailandensis*. These studies reveal that human GBP1 uniquely functions as an intracellular “glue trap,” inhibiting the cytosolic movement of normally actin-propelled Gram-negative bacteria. In response to this powerful human defense program, *S. flexneri* has evolved an effective counterdefense to restrict GBP1 recruitment.

## IMPORTANCE

Several pathogenic bacterial species evolved to invade, reside in, and replicate inside the cytosol of their host cells. One adaptation common to most cytosolic bacterial pathogens is the ability to coopt the host’s actin polymerization machinery in order to generate force for intracellular movement. This actin-based motility enables Gram-negative bacteria, such as *Shigella* species, to propel themselves into neighboring cells, thereby spreading from host cell to host cell without exiting the intracellular environment. Here, we show that the human protein GBP1 acts as a cytosolic “glue trap,” capturing cytosolic Gram-negative bacteria through a unique protein motif and preventing disseminated infections in cell culture models. To escape from this GBP1-mediated host defense, *Shigella* employs a virulence factor that prevents or dislodges the association of GBP1 with cytosolic bacteria. Thus, therapeutic strategies to restore GBP1 binding to *Shigella* may lead to novel treatment options for shigellosis in the future.

## INTRODUCTION

Cell-autonomous immunity describes the ability of a single cell to defend itself against intracellular pathogens and constitutes an essential branch of the immune system (1, 2). Cell-autonomous immunity in vertebrates is often orchestrated by interferon (IFN)-stimulated genes (ISGs) (2). Among the most robustly expressed ISGs are those encoding dynamin-like guanylate binding proteins (GBPs) (3–5). GBPs control intrinsic antiviral, antiprotozoan, and antibacterial immunity, are highly expressed in inflamed tissue, and can be predictive of infectious disease progressions (5–10). Since their discovery, seven human *GBP* orthologs and one pseudogene have been identified. The *GBP* genes are located within one gene cluster on chromosome 1 (11). Other vertebrate genomes contain comparable numbers of *GBP* orthologs; e.g., mice possess 11 genes in addition to 2 pseudogenes (12). Human and mouse GBPs share a high degree of homology, with the most conserved region found within their N-terminal G domains. However, GBP protein family members are highly divergent from each other at their very C-terminal ends, both within and across different vertebrate species (11). The functional consequence of this C-terminal amino acid sequence variability has not been previously explored.

To exert many of their antimicrobial functions, GBPs specifically associate with intracellular microbes residing in the host cell cytosol or at pathogen-occupied supramolecular structures, which include viral replication complexes (10) and pathogen-containing vacuoles (3–5). Following pathogen recognition, GBPs are thought to deliver antimicrobial host factors to pathogen-containing vacuoles and to bacteria residing in the host cell cytosol, thereby enabling the execution of distinct defense pathways which include membranolytic destruction of cytosolic bacteria (13, 14), capture of microbes within degradative autolysosomes (7), and activation of inflammasomes (13–19). These studies clearly demonstrated the importance of GBPs in host defense against a broad spectrum of pathogens in a vertebrate host. However, because these previous studies were conducted almost exclusively with mouse models, it remains to be determined whether human GBPs execute functions comparable to those of their murine counterparts.

In this study, we systematically tested all seven members of the human GBP protein family for their ability to colocalize with the cytosolic bacterial pathogens *Listeria monocytogenes*, *Shigella flexneri*, and *Burkholderia thailandensis*. All of these bacterial species are equipped with the ability to coopt the host actin polymerization machinery for actin-based cytosolic motility and cell-to-cell spread (20). While we failed to detect any colocalization between human GBPs and the Gram-positive bacterium *L. monocytogenes*, we found that GBP1, independently of other human GBP paralogs, targeted both of the Gram-negative bacteria *B. thailandensis* and *S. flexneri*. This specific interaction between GBP1 and bacteria, which is determined by a unique C-terminal triple-arginine motif, inhibits actin tail formation of GBP1-decorated bacteria, resulting in a reduction of bacterial cell-to-cell spread. We further observed that GBP1 targeted the non-human-adapted microbe *B. thailandensis* more efficiently than the human-adapted pathogen *S. flexneri*, leading us to hypothesize that *S. flexneri* actively interferers with GBP1 function. In agreement with this hypothesis and confirming similar results published during the preparation of this paper (21, 22), we identified the bacterial ubiquitin E3 ligase IpaH9.8 as an *S. flexneri* virulence factor that interferes with GBP1 recruitment to this professional cytosolic bacterium. Thus, this study provides a novel understanding of the role of human GBPs in immunity to bacterial pathogens and also defines a virulence strategy employed by a human-adapted microbe to escape from GBP1-regulated host defense.

## RESULTS

### Human GBP1 colocalizes with cytosolic *S. flexneri* and *B. thailandensis*.

**FIG 1:**
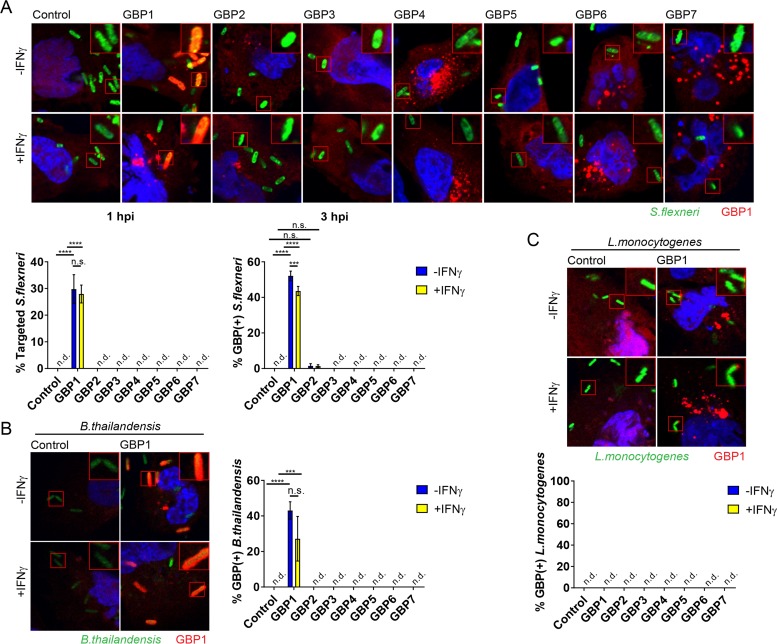
Ectopically expressed human GBP1 colocalizes with Gram-negative *S. flexneri* and *B. thailandensis* but not Gram-positive *L. monocytogenes* in human A549 cells. A549 cells were transfected with the indicated GBP paralogs fused to mCherry at their N termini or transfected with the mCherry control. Cells were primed with IFN-γ at 200 U/ml overnight or left untreated. (A) Cells were infected with GFP-positive *S. flexneri* at a multiplicity of infection (MOI) of 50, and microscopy images were taken at 1 and 3 hpi (1-hpi images are shown). (B) Cells were infected with GFP-positive *B. thailandensis* at an MOI of 100, and images were acquired at 8 hpi. (C) Cells were infected with GFP-positive *L. monocytogenes* at an MOI of 5 and monitored at 1 hpi and 3 hpi (1-hpi image and 1-h data are shown). (A to C) Combined data from 3 independent experiments are shown. Per experiment, >200 bacteria were scored in transfected, i.e., mCherry-positive, cells. Error bars indicate SEM. Significance was determined by two-way ANOVA. ***, *P* < 0.001; ****, *P* < 0.0001; n.s., nonsignificant; n.d., not detectable.

Most known GBP-mediated antimicrobial functions require that GBPs directly localize to intracellular microbes or their surrounding vacuoles (3–5). Based on this premise, we screened the entire set of human GBPs (GBP1 to -7) as ectopically expressed mCherry N-terminal fusion proteins in the human epithelial lung carcinoma cell line A549 for colocalization with the cytosolic bacterial pathogen *S. flexneri*. Expression of each fusion protein was detectable by Western blotting (Fig. S1) and by immunofluorescence assay (Fig. 1A). Unexpectedly, only a single member, GBP1, associated with cytosolic *S. flexneri*, as assessed at 1 and 3 h postinfection (hpi) in either naive or gamma interferon (IFN-γ)-primed A549 cells (Fig. 1A). Association of GBP1 with individual *S. flexneri* bacteria was observed as early as 10 min after host cell invasion (data not shown and Movie S1 in the supplemental material). Similarly, we observed that GBP1 was the sole human GBP family member to colocalize with the cytosolic Gram-negative bacterium *B. thailandensis* in either naive or IFN-γ-primed A549 cells (Fig. 1B). Unlike with the observed targeting of GBP1 to these Gram-negative bacteria, we failed to detect any colocalization of any human GBP paralog with *L. monocytogenes*, a cytosolic Gram-positive pathogen (Fig. 1C).

10.1128/mBio.01979-17.1 FIG S1  Expression of N-terminal mCherry-GBP fusion proteins in HEK 293T cells. HEK 293T cells were transiently transfected with mCherry-tagged human GBPs. Cells were lysed in RIPA buffer approximately 24 h posttransfection and probed with anti-GBP1 and anti-GAPDH antibodies. Download FIG S1, JPG file, 1.2 MB. Copyright © 2017 Piro et al. 2017 Piro et al. This content is distributed under the terms of the Creative Commons Attribution 4.0 International license.

10.1128/mBio.01979-17.8 MOVIE S1  *GBP1*^KO^ HeLa cells transfected with mCherry-GBP1 were infected with poly-d-lysine-pretreated *S. flexneri* expressing GFP at an MOI of 10. Images were collected every 30 s for 3 h, beginning at 10 min postinfection. Download MOVIE S1, AVI file, 6.8 MB. Copyright © 2017 Piro et al. 2017 Piro et al. This content is distributed under the terms of the Creative Commons Attribution 4.0 International license.

### Targeting of GBP1 to *S. flexneri* is dependent on its functional G domain, CaaX box, and C-terminal triple-arginine motif.

**FIG 2:**
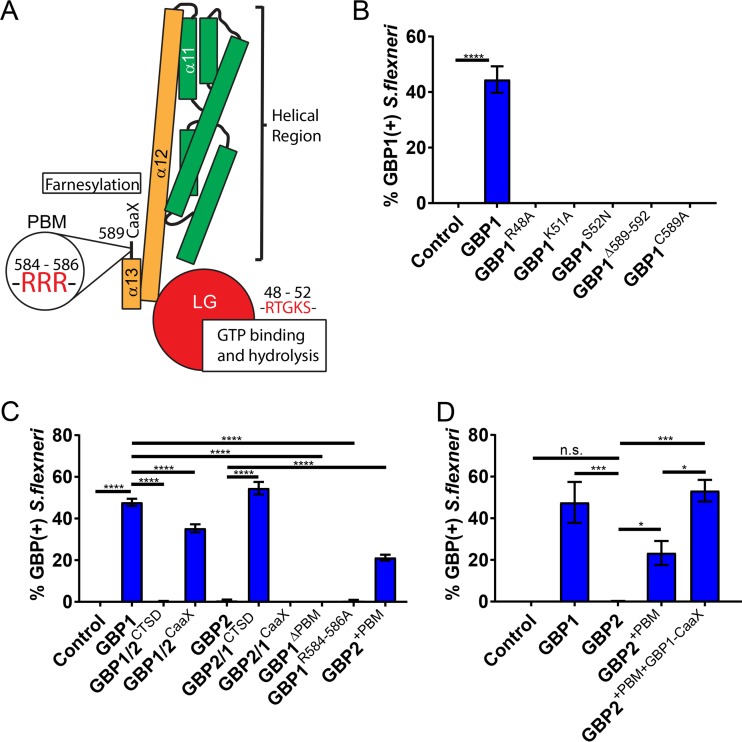
Targeting of GBP1 to *S. flexneri* is dependent on its functional G domain, its CaaX box, and a C-terminal triple-arginine motif. (A) Schematic depiction of critical protein motifs, domains, and specific residues within the structure of human GBP1. (B) A549 cells were transfected with the mCherry construct (Control), mCherry-tagged wild-type GBP1, and mCherry-tagged GBP1 variants defective in GTP hydrolysis (R48A), nucleotide binding (K51A and S52N), or farnesylation (Δ589–592 and C589A), as indicated. Cells were infected with GFP^+^ *S. flexneri* at an MOI of 50 and assessed for colocalization with mCherry at 3 hpi inside mCherry-expressing cells. (C and D) HEK 293T cells were transfected with the mCherry construct (Control), mCherry-tagged wild-type constructs (GBP1 and GBP2), and chimeric (GBP1/2^CTSD^, GBP1/2^CaaX^, GBP2/1^CTSD^, GBP2/1^CaaX^), deletion mutant (GBP1Δ^PBM^), triple mutant (R584–586A), or insertion mutant (GBP2^+PBM^, GBP2^+PBM+GBP1-CaaX^) constructs. Cells were infected with GFP^+^ *S. flexneri* at an MOI of 50 and assessed for colocalization with mCherry at 3 hpi inside mCherry-expressing cells. CTSD, C-terminal subdomain; PBM, polybasic protein motif. Combined data from 3 independent experiments are shown. Per experiment, >200 bacteria were scored. Error bars indicate SEM. Significance was determined by one-way ANOVA relative to results for GBP1 (B) or as indicated (C and D). *, *P* < 0.05; ***, *P* < 0.001; ****, *P* < 0.0001; n.s., nonsignificant.

To determine which protein motifs and properties of GBP1 (Fig. 2A) render it uniquely capable of detecting cytosolic Gram-negative bacteria, we generated and screened a large set of GBP1 mutant variants for colocalization with cytosolic *S. flexneri*. Mutant variants previously established as being defective for GTP hydrolysis (GBP1^R48A^) or nucleotide binding (GBP1^K51A^ and GBP1^S52N^) (23, 24) failed to associate with *S. flexneri* (Fig. 2B). These findings were expected, because GTP binding and hydrolysis are required for GBP1 dimerization, protein polymerization, and membrane binding (23–28). We also found that targeting to *S. flexneri* was dependent on the C-terminal CaaX box of GBP1 (Fig. 2B). This finding was also expected, as CaaX box-dependent prenylation of GBP1 provides a lipid anchor critical for the membrane association of GBP1 (27–29).

Both GBP1 and GBP2 contain highly homologous N-terminal G domains and carry C-terminal CaaX boxes (11), yet only GBP1 efficiently associates with *S. flexneri* in A549 cells (Fig. 1A). We therefore generated protein chimeras between GBP1 and GBP2 to map the motif that uniquely directs GBP1 to cytosolic bacteria. Most of the divergence between GBP1 and GBP2 sequences is found within the C-terminal subdomain (CTSD) consisting of α-helices α12 and α13, a short flexible region, and the CaaX box. We therefore swapped the CTSDs of GBP1 and GBP2 to generate two complementary chimeric proteins and found that the GBP1- but not the GBP2-derived CTSD determined protein targeting to *S. flexneri* (Fig. 2C). Swapping the CaaX boxes on the other hand only moderately reduced GBP1 targeting to bacteria (Fig. 2C), indicating the existence of an additional motif within the GBP1 CTSD critical for bacterial recognition. Within the flexible region of CTSD, we identified a GBP1-specific polybasic protein motif (PBM), a 6-amino-acid stretch containing 5 basic residues (KMRRRK). Deletion of these 6 residues or mutation of the triple-arginine cassette to triple alanine abrogated colocalization of GBP1 with *S. flexneri* (Fig. 2C). In a complementary approach, we found that insertion of the GBP1 PBM between GBP2 residues 586 and 587 (GBP2^+PBM^) was sufficient to drive significant targeting of GBP2 to *S. flexneri* (Fig. 2C). Swapping the GBP2 CaaX box with that of GBP1 (GBP2^+PBM+GBP1-CaaX^) further improved the bacterium-targeting efficiency such that GBP2^+PBM+GBP1-CaaX^ is recruited to *Shigella* at levels comparable to those of wild-type GBP1 (Fig. 2D). These data demonstrate that the C-terminal PBM of GBP1 is essential and sufficient to equip both GBP1 and GBP2 with the ability to detect cytosolic *S. flexneri* and that the GBP1 CaaX box further improves targeting efficiency.

### Triple-arginine motif controls delivery of GBP1 to sterilely damaged vacuoles.

**FIG 3:**
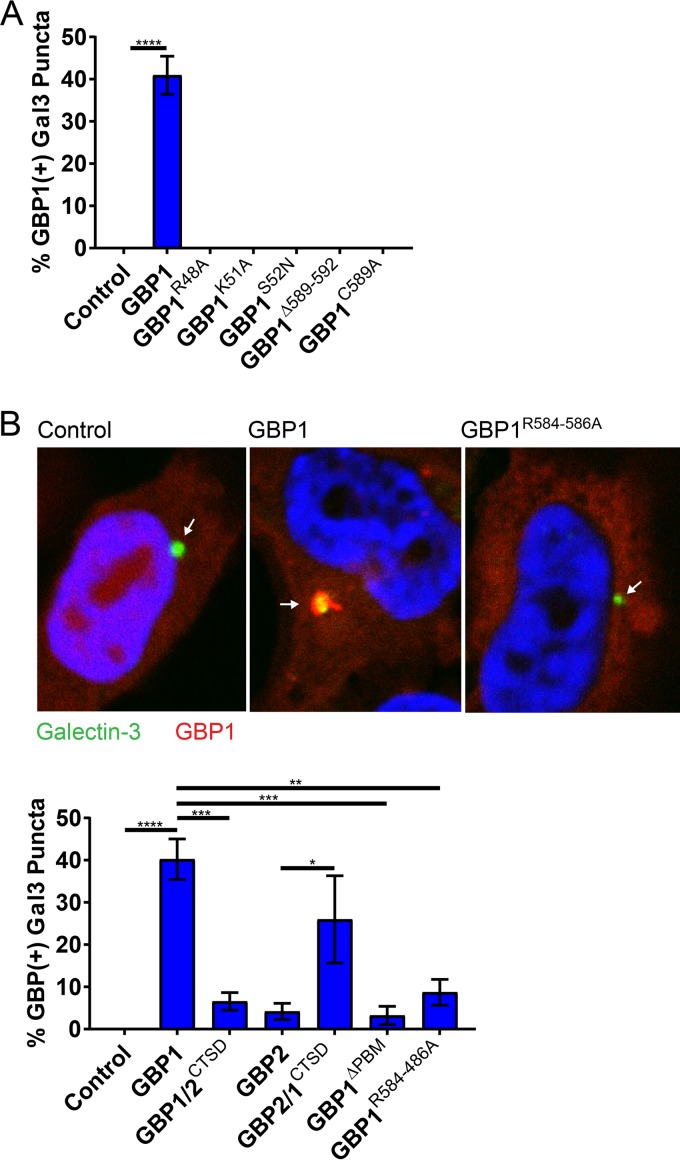
Localization of GBP1 to damaged endosomes is dependent on its triple-arginine motif. HEK 293T cells stably expressing YFP–galectin-3 (Gal3) were transiently transfected with the indicated expression constructs and approximately 1 day later treated with calcium phosphate precipitates to induce endosomal damage leading to YFP-Gal3 puncta formation. (A and B) Combined data from 3 independent experiments are shown. Per independent experiment, >400 YFP-Gal3 puncta were scored for GBP1 colocalization in mCherry-positive cells. Error bars denote SEM. One-way ANOVA was used to assess significance. **, *P* < 0.01; ***, *P* < 0.001; ****, *P* < 0.0001; n.s., nonsignificant. (B) Arrows within representative images point to galectin-3-positive vacuoles.

To identify possible molecular targets for the C-terminal PBM of GBP1, we followed up on our previous published observation that GBP1, but none of its human paralogs, detects sterilely damaged endogenous vacuoles in human embryonic kidney 293T (HEK 293T) cells (30). The disruption of vacuoles leads to the cytosolic exposure of glycans that are normally confined to the vacuolar lumen. These exposed sugars then prompt the recruitment of the β-galactoside-binding lectin galectin-3 as an established marker for loss of vacuolar integrity (31). To determine whether the PBM of GBP1 could promote the recognition of glycans, we tested its role in the delivery of GBP1 to ruptured vesicles. We first determined whether the structural requirements for the delivery of GBP1 to ruptured endosomes generally resembled those for the targeting of GBP1 to bacteria. In support of this premise, we observed that delivery of GBP1 to damaged endosomes was dependent on its functional G domain and CaaX box (Fig. 3A). We further noticed that the CTSD of GBP1 was essential for the delivery of GBP1 and sufficient to promote the recruitment of chimeric GBP2 protein to damaged vesicles (Fig. 3B). Deletion of PBM or mutation of the triple-arginine motif led to substantially reduced colocalization between galectin-3-marked vacuoles and GBP1 (Fig. 3B), indicating a functional role for the triple-arginine motif in the delivery of GBP1 to disrupted vesicles. These observations suggested that the triple-arginine motif of GBP1 directly or indirectly detects glycans.

### *S. flexneri* mutants lacking O antigen are targeted infrequently by GBP1.

**FIG 4:**
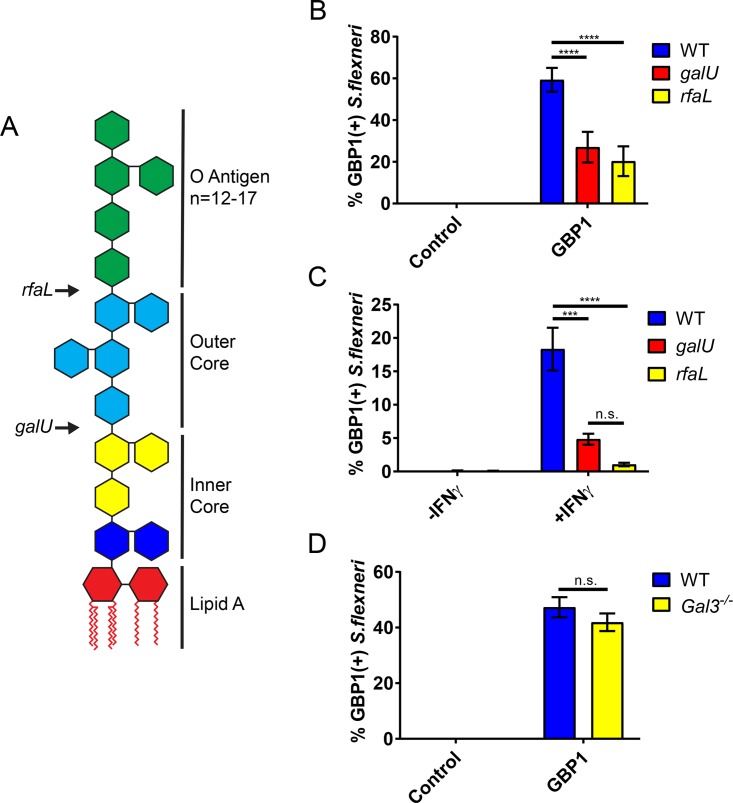
Bacterial targeting of GBP1 is diminished for *S. flexneri* rough mutants. (A) Arrows indicate sites of truncation of the LPS structure resulting from the loss-of-function mutations in the bacterial genes *galU* and *rfa*. HEK 293T cells were transfected with mCherry-GBP1 or the mCherry control (B), whereas HeLa cells were left untransfected (C). Following infection with the wild type or the indicated *S. flexneri* mutants at an MOI of 50, colocalization of ectopically expressed GBP1 (B) or endogenous GBP1 (C) was determined. Combined data from at least 3 independent experiments are shown. Per experiment, >400 bacteria were scored per experimental condition. (D) Primary wild-type (WT) (C57BL/6J) and *galectin-3*^−/−^ MEFs were transiently transfected with expression constructs for mCherry-GBP1 or an mCherry control and subsequently infected with GFP^+^ *S. flexneri* at an MOI of 50. Cells were fixed at 3 hpi, and the percentage of GBP1-positive bacteria was determined by microscopy. Graphs represent the mean percentages of GBP1-positive bacteria from three independent experiments. Error bars represent SEM. Two-way ANOVA was used to assess statistical significance. ***, *P* < 0.001; ****, *P* < 0.0001; n.s., nonsignificant.

Because GBP1 detects broken vacuoles that are marked by cytosolically exposed polysaccharides normally confined to the vacuolar lumen, we speculated that bacterial surface-exposed polysaccharides similarly underlie the recruitment of GBP1 to *S. flexneri*. We thus investigated whether GBP1 either directly or indirectly detects lipopolysaccharide (LPS), the main building block of the Gram-negative bacterial envelope. Because O antigen forms the outward sugar portion of LPS on the surfaces of bacteria, we tested GBP1 recruitment to the O-antigen-deficient *galU* and *rfaL* “rough” *S. flexneri* mutants, which enter the host cell cytosol of nonpolarized epithelial cells at comparable frequencies (32). We found that colocalization of ectopically expressed (Fig. 4B) or endogenous (Fig. 4C) GBP1 with rough *S. flexneri* mutants was substantially diminished relative to that of GBP1 targeting wild-type *S. flexneri*, indicating that O-antigen recognition plays an important role in GBP1’s recognition of cytosolic Gram-negative bacteria.

We previously demonstrated that galectin-3 interacts with a subset of murine GBPs and promotes their recruitment to *Legionella*- as well as *Yersinia*-containing vacuoles (30). Because galectin-3 binds to O antigen as well as the inner core of LPS (33), we hypothesized that galectin-3 promotes the delivery of GBP1 to the Gram-negative bacterium *S. flexneri*. However, we observed that ectopically expressed GBP1 colocalized with *S. flexneri* with comparable frequencies in wild-type and galectin-3-deficient mouse embryonic fibroblasts (MEFs) (Fig. 4D). Together, these data suggest that GBP1 detects cytosolic *S. flexneri* in an O-antigen-dependent but galectin-3-independent manner.

### GBP1-decorated *S. flexneri* cells replicate within intracellular bacterial clusters.

**FIG 5:**
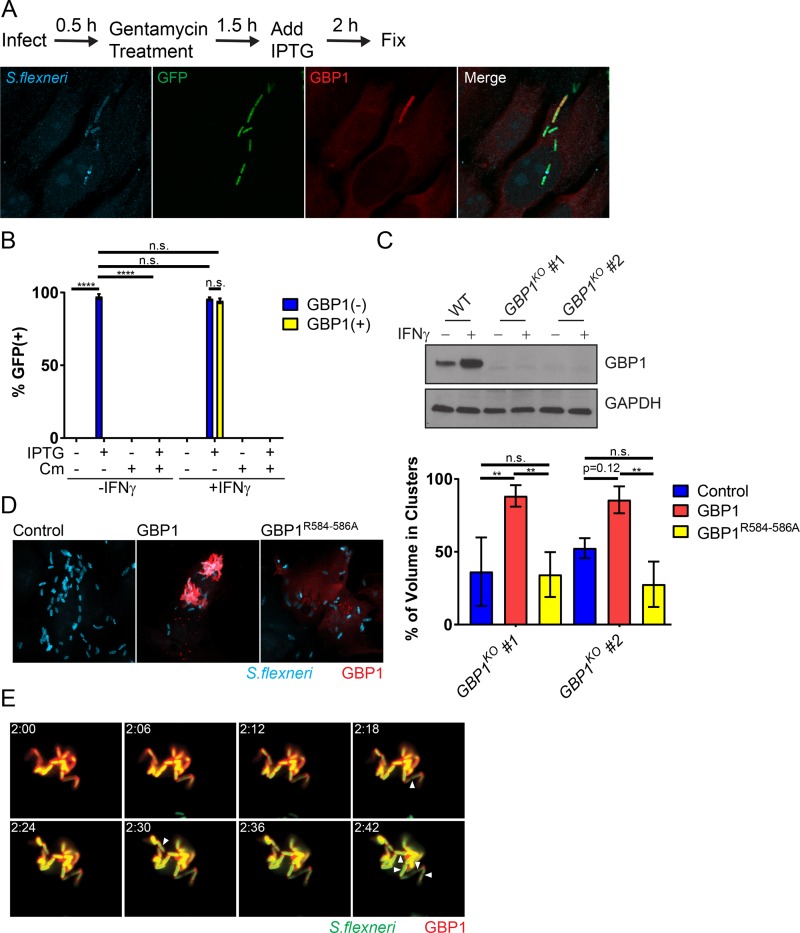
GBP1-tagged *S. flexneri* cells replicate within intracellular bacterial aggregates. (A) IFN-γ-primed and unprimed HeLa cells were infected at an MOI of 50 with *S. flexneri* carrying an IPTG-inducible GFP reporter plasmid. IPTG and, where indicated, also chloramphenicol (Cm) were added to the culture medium at 2 hpi. At 4 hpi, cells were fixed and stained with anti-LPS (blue) and anti-GBP1 (red). A representative image of infected, IFN-γ-primed HeLa cells is shown. (B) GFP expression was used as a proxy for bacterial viability. Combined data from 3 independent experiments as described for panel A are shown. (C) Protein lysates from IFN-γ-primed parental HeLa (WT) and two independent *GBP1*^KO^ clones were transferred to membranes and probed with anti-GBP1 and anti-GAPDH antibodies. (D) Two independent *GBP1*^KO^ HeLa cell clones were transfected with wild-type GBP1 or GBP1^R584–586A^ mCherry fusion proteins and then infected with *S. flexneri* at an MOI of 50. Bacteria were visualized with anti-LPS (blue) immunostaining. Cluster analysis was performed as described in Materials and Methods, and the percentage of total bacterial volume occupied by clusters of greater than three bacteria was determined in mCherry-positive cells using the ImageJ 3D-OC plugin. The graph depicts the combined data from three independent experiments. Error bars represent SEM. (E) *GBP1*^KO^ HeLa cells were transfected with mCherry-GBP1 and infected with poly-d-lysine-pretreated *S. flexneri* at an MOI of 10. Cells were infected for 30 min, washed, and then placed in phenol red-free DMEM. Video microscopy was begun at 2 hpi (2:00 a.m.). Images of 6-min intervals between 2:00 a.m. and 2:42 are shown. Arrowheads point to bacteria that have undergone cell division. Two-way ANOVA was used to assess statistical significance. ****, *P* < 0.0001; n.s., nonsignificant.

Previous work demonstrated the lytic destruction of GBP-bound cytosolic bacteria in mouse cells (13, 14). To assess whether recruitment of human GBP1 to *S. flexneri* would similarly drive bactericidal effects, we used an IPTG (isopropyl-β-d-thiogalactopyranoside)-inducible green fluorescent protein (GFP) reporter system as a bacterial viability indicator. In these experiments, we infected HeLa cells with reporter-positive *S. flexneri* and then induced GFP expression with IPTG at 2 hpi and stained cells for endogenous GBP1 and LPS at 4 hpi (Fig. 5A). As expected, inhibition of bacterial translation with chloramphenicol treatment at 2 hpi blocked detectable GFP expression at 4 hpi (Fig. 5B), thus validating the reporter system. We then primed HeLa cells with IFN-γ to induce endogenous GBP1 expression and to assess the effect of GBP1 targeting on bacterial viability. We found that GBP1-targeted and -untargeted bacteria were GFP positive at comparable frequencies, indicating that GBP1 recruitment to *S. flexneri* fails to promote bacterial killing at this time point (Fig. 5B). Instead, we noticed that GBP1-positive bacteria form intracellular clusters. To measure clustering, we developed an automated scoring matrix (Fig. S2), as described in Materials and Methods. To determine whether GBP1 might be responsible for this clustering effect, we generated HeLa cells with *GBP1* knocked out (*GBP1*^KO^) (Fig. 5C). Following IFN-γ priming to induce expression of GBPs, we observed reduced bacterial clustering in two independent *GBP1*^KO^ clonal cell lines compared to the clustering in the parental HeLa cells (Fig. 5D). Bacterial clustering was restored in *GBP1*^KO^ cells by ectopically expressing wild-type GBP1 but not a triple-arginine mutant (Fig. 5D). Furthermore, we found that GBP1-decorated, clustered bacteria not only remained viable but continued to divide (Fig. 5E; Movie S2). Thus, our observations indicate that triple-arginine-dependent delivery of GBP1 to cytosolic *S. flexneri* promotes bacterial clustering but fails to execute bactericidal or bacteriostatic activities.

10.1128/mBio.01979-17.2 FIG S2  A three-dimensional object counter is used to identify bacterial clusters. Cells were infected with *S. flexneri* at an MOI of 50 and fixed at 3 hpi. Bacteria were then stained with an anti-LPS antibody (blue). Shown here is a representative maximum projection of Z-stacks containing LPS signals. Yellow outlines depict nonclustered objects, and red outlines depict clustered objects. Download FIG S2, JPG file, 0.5 MB. Copyright © 2017 Piro et al. 2017 Piro et al. This content is distributed under the terms of the Creative Commons Attribution 4.0 International license.

10.1128/mBio.01979-17.9 MOVIE S2  *GBP1*^KO^ HeLa cells transfected with mCherry-GBP1 were infected with poly-d-lysine-pretreated *S. flexneri* expressing GFP at an MOI of 10. Images were collected every 90 s for 45 min, beginning at 190 min postinfection. Download MOVIE S2, AVI file, 0.8 MB. Copyright © 2017 Piro et al. 2017 Piro et al. This content is distributed under the terms of the Creative Commons Attribution 4.0 International license.

### GBP1 inhibits actin-based motility in a triple-arginine-motif-dependent manner.

**FIG 6:**
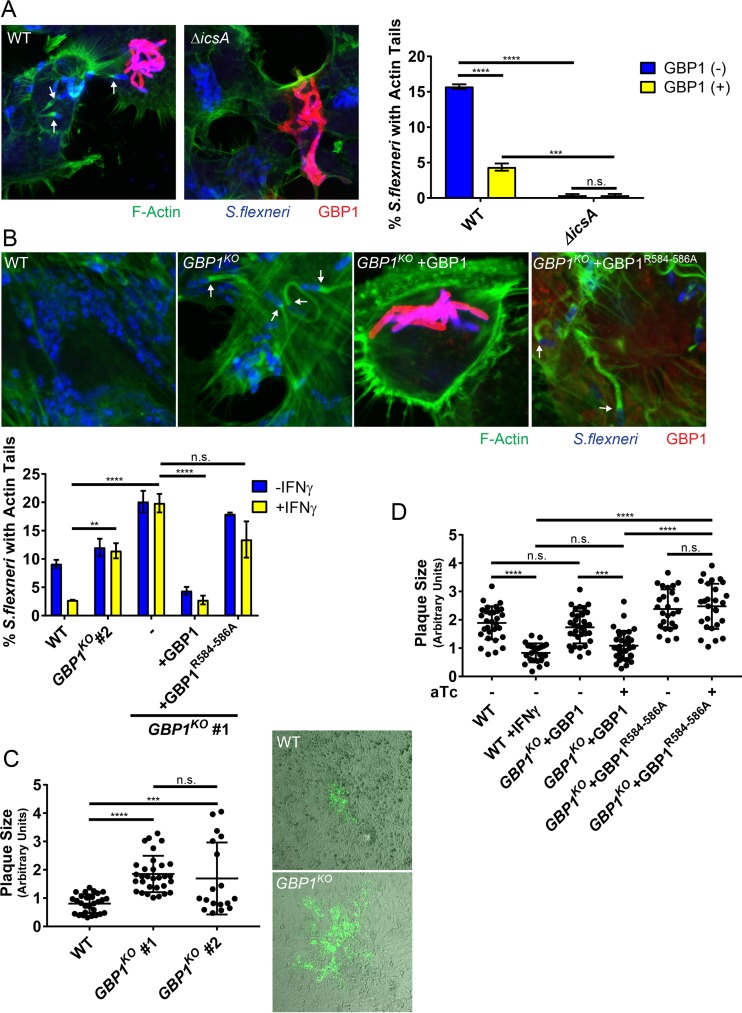
GBP1 restricts actin tail formation and cell-to-cell spread via its triple-arginine motif. (A) HEK 293T cells were transfected with mCherry-GBP1 and then infected with wild-type (WT) or *ΔicsA S. flexneri* at an MOI of 50. Confocal images were taken at 3 hpi and analyzed for actin tail formation as described in Materials and Methods. Arrows point at actin tails. Data are combined from 3 independent experiments. Per experiment and condition, >550 bacteria were examined in mCherry-positive cells. (B) Parental HeLa-Cas9 (WT) and *GBP1*^KO^ cells were transfected with mCherry-GBP1 or mCherry-GBP1^R584–586A^ as indicated and then primed with IFN-γ overnight and infected with *S. flexneri* at an MOI of 50. Cells were fixed and stained at 3 hpi, and representative images are shown. Arrows point at actin tails. Bacterial actin tail formation inside mCherry-positive cells was quantified, and combined data from 3 independent experiments are shown. (C) Plaque assays of IFN-γ-primed cells were performed as described in Materials and Methods, and combined data from 3 independent experiments as well as representative images are shown. (D) HeLa-Cas9 (WT) cells and the *GBP1*^KO^ clone (#1) with stably integrated pInducer-GBP1 or pInducer-GBP1^R584–586A^ expression constructs where either primed with IFN-γ or stimulated with 1 µg/ml aTc, as indicated. Combined data from 3 independent experiments are shown. Error bars depict SEM (A to C) or standard deviations (SD) (D). Two-way (A to B) and 1-way (C to D) ANOVAs were used to determine statistical significance. **, *P* < 0.01; ***, *P* < 0.001; ****, *P* < 0.0001; n.s., nonsignificant.

GBP1-decorated *S. flexneri* organisms cluster intracellularly and therefore phenocopy *S. flexneri* Δ*icsA* mutants (Fig. 6A) that lack the ability to form actin tails and hence are nonmotile inside the host cell cytosol (34). Thus, we hypothesized that GBP1 blocks *S. flexneri* actin tail formation. In support of this hypothesis, we observed a marked reduction in actin tails associated with GBP1-tagged versus -untagged *S. flexneri* in 293T cells expressing mCherry-GBP1 (Fig. 6A). To complement these GBP1 overexpression studies, we scored actin tail-positive bacteria in wild-type versus *GBP1*^KO^ HeLa cells. We found that IFN-γ priming reduced the number of bacteria with actin tails in wild-type but not in *GBP1*^KO^ cells (Fig. 6B). Complementation of *GBP1*^KO^ cells with wild-type GBP1 but not the triple-arginine mutant (GBP1^R584–586A^) dramatically reduced the number of actin tail-positive bacteria to levels observed in IFN-γ-primed wild-type cells (Fig. 6B). Because actin-based motility is required for bacterial cell-to-cell spread, we measured the impact of IFN-γ-induced GBP1 expression on bacterial dissemination by performing bacterial plaque assays. As expected, GBP1-mediated inhibition of actin tail formation correlated with a GBP1-mediated block in cell-to-cell spread (Fig. 6C). Inhibition of cell-to-cell spread was restored in *GBP1*^KO^ cells complemented with the wild type but not the triple-arginine mutant (GBP1^R584–586A^) (Fig. 6D), indicating that GBP1 targeting to bacteria is required for inhibition of bacterial cell-to-cell spread.

### Endogenous GBP1 associates with *S. flexneri* and recruits additional GBP paralogs in HeLa but not in A549 cells.

**FIG 7:**
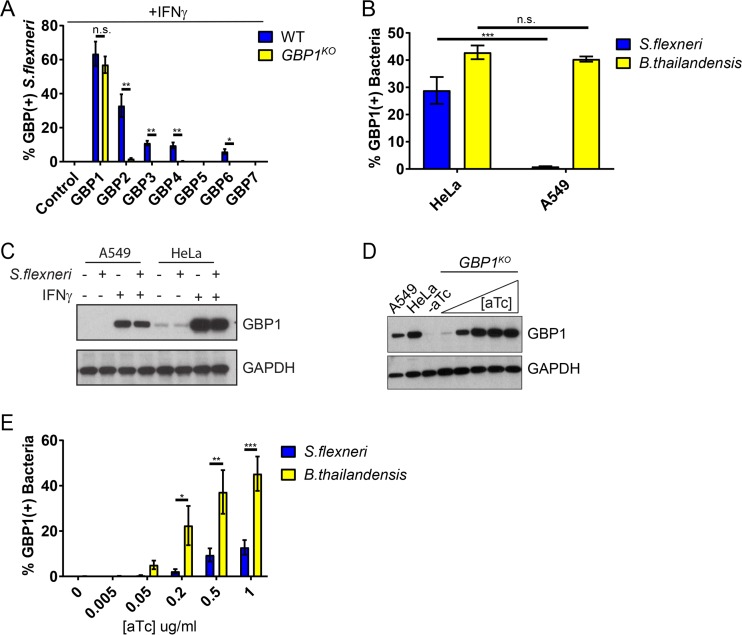
Endogenous GBP1 colocalizes with *S. flexneri* and recruits additional GBP paralogs in HeLa but not in A549 cells. (A) HeLa-Cas9 (WT) and *GBP1*^KO^ cells were transfected with the indicated mCherry-GBP expression constructs and primed with IFN-γ overnight. Cells were infected with GFP^+^ *S. flexneri* at an MOI of 50 and fixed at 3 hpi, and mCherry-positive cells were analyzed for bacterial colocalization with GBPs. The combined data from 3 independent experiments are shown. Per experiment and condition, >300 bacteria were scored. Error bars denote SEM, and Student’s *t* tests were used to determine statistical significance. (B) IFN-γ-primed HeLa and A549 cells were infected with GFP^+^ *S. flexneri* at an MOI of 50 or with GFP^+^ *B. thailandensis* at an MOI of 100 and fixed and stained with anti-GBP1 at 3 hpi and 8 hpi, respectively. Combined data are from 3 independent experiments (>600 bacteria were counted per experiment) are shown. Error bars represent SEM, and two-way ANOVA was used to assess significance. (C) Expression of endogenous GBP1 in IFN-γ-primed HeLa and A549 cells was assessed by Western blotting. (D) *GBP1*^KO^ HeLa cells with stably integrated pInducer-GBP1 were stimulated overnight with the indicated concentrations of aTc, and protein lysates were subjected to Western blotting. Protein lysates from IFN-γ-primed A549 and HeLa-Cas9 cells were included for comparison. (E) *GBP1*^KO^ HeLa cells with stably integrated pInducer-GBP1 were stimulated overnight with the indicated concentrations of aTc and then infected with GFP^+^ *S. flexneri* (MOI of 50) for 3 h or GFP^+^ *B. thailandensis* (MOI 100) for 8 h and analyzed for bacterial colocalization with endogenous GBP1. Combined data from 3 independent experiments (>400 bacteria were counted per experiment) are shown. Error bars represent SEM. *, *P* < 0.05; **, *P* < 0.01; ***, *P* < 0.001; n.s., nonsignificant.

GBP1 frequently forms heterodimers with other members of the GBP family, such as GBP2 (3–5). To determine whether GBP1 recruits other GBP paralogs to cytosolic *S. flexneri*, we ectopically expressed mCherry-tagged human GBP1 to -7 in wild-type and *GBP1*^KO^ HeLa cells and monitored for subcellular colocalization of each with cytosolic *S. flexneri*. We found that ectopically expressed GBP2 and, to a lesser extent, GBP3, GBP4, and GBP6 colocalized with *S. flexneri* in HeLa cells in a GBP1-dependent manner (Fig. 7A). This finding was curious, as we had not observed any colocalization of these GBP paralogs with *S. flexneri* in wild-type A549 cells (Fig. 1A). We therefore monitored the subcellular localization of endogenous GBP1 in A549 cells and found that endogenous GBP1 targeted *S. flexneri* in HeLa but not in A549 cells (Fig. 7B), which correlated with reduced expression of GBP1 protein in A549 compared to in HeLa cells (Fig. 7C). While endogenous GBP1 failed to decorate *S. flexneri* in A549 cells, we noticed that in the same cell line, about 40% of cytosolic *B. thailandensis* bacteria stained positive for endogenous GBP1 (Fig. 7B). These data suggested that *B. thailandensis* is more susceptible to GBP1 targeting than *S. flexneri*. To test this hypothesis, we generated a titratable, i.e., anhydrotetracycline (aTc)-inducible, expression system for GBP1 (Fig. 7D). As expected, higher GBP1 expression levels promoted more-frequent colocalization with either bacterium. However, the overall frequency of GBP1 colocalization was significantly higher for *B. thailandensis* than for *S. flexneri* (Fig. 7E). These data indicated that *S. flexneri* is more resistant to GBP1 targeting than *B. thailandensis* and therefore suggested that *S. flexneri* actively interferes with cytosolic detection by GBP1.

### IpaH9.8 blocks GBP1 recruitment and GBP1-mediated inhibition of actin-based motility.

**FIG 8:**
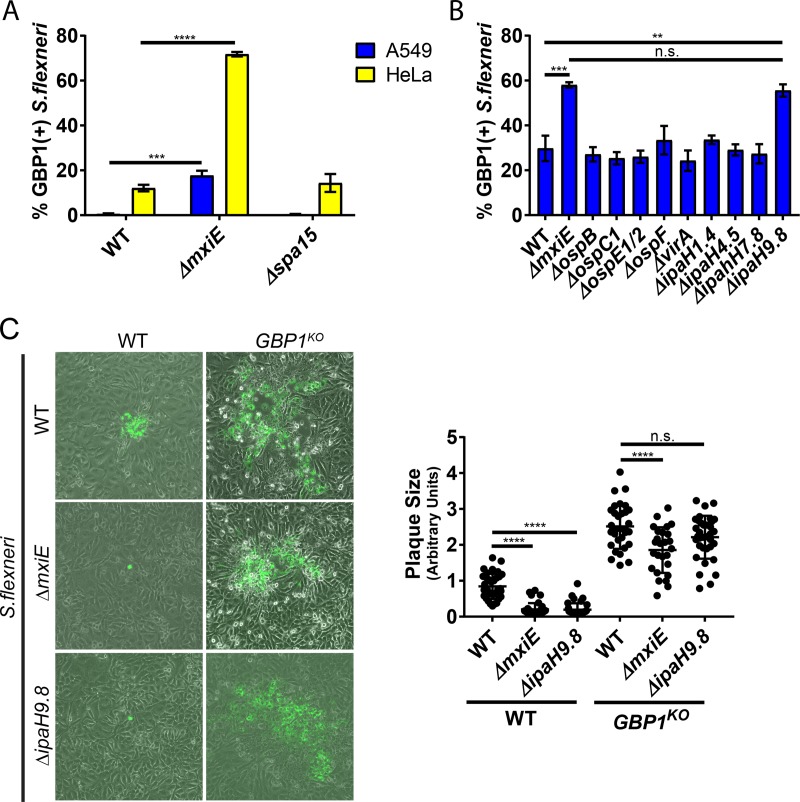
IpaH9.8 blocks GBP1 recruitment and GBP1-mediated inhibition of actin-based motility. (A) IFN-γ-primed HeLa and A549 cells were infected with the indicated *S. flexneri* strains at an MOI of 50 and at 3 hpi processed and analyzed for colocalization with endogenous GBP1. Data are combined from 3 independent experiments scoring 600 bacteria per experiment. One-way ANOVA was used to determine significance. (B) Experiments were conducted as described for panel A with the *S. flexneri* mutant strains, as listed. Two-way ANOVA was used to determine the significance of combined data from 3 independent experiments. (C) IFN-γ-primed HeLa-Cas9 and derived *GBP1*^KO^ cells were infected with the indicated *S. flexneri* mutant strains. Representative images of plaques are shown. Combined data from 3 independent experiments are depicted. Error bars indicate SD. Two-way ANOVA was used to determine significance. **, *P* < 0.01; ***, *P* < 0.001; ****, *P* < 0.0001; n.s., nonsignificant.

To account for our observations, we hypothesized that an effector secreted by the *Shigella* type 3 secretion system (T3SS) interferes with GBP1 targeting to cytosolic bacteria. To test this hypothesis, we monitored colocalization of GBP1 with two *S. flexneri* mutants deficient for the secretion of distinct subsets of type III effectors. A bacterial mutant lacking Spa15, the chaperone required for the secretion of the effectors IpaA, IpgB1, IpgB2, OspB, OspC1, OspC2, OspC3, OspD1, and OspD2 (35–38), was targeted with the same efficiency as in wild-type *S. flexneri* (Fig. 8A). However, *Shigella* Δ*mxiE*, a strain lacking the transcription factor that controls expression of second-phase *S. flexneri* effectors, including all IpaH effectors as well as OspB, OspC1, OspE1, OspE2, OspF, OspG, and VirA (39–41), colocalized more frequently with GBP1 than did wild-type *S. flexneri* in both A549 and HeLa cells (Fig. 8A). These findings indicated that one or more *mxiE*-dependent T3SS effectors interfered with GBP1 function. To identify this virulence factor, we screened *S. flexneri* mutants deficient in individual *mxiE*-dependent T3SS effectors encoded on the *S. flexneri* virulence plasmid and found that the Δ*ipaH9*.*8* strain mimicked the Δ*mxiE* phenotype (Fig. 8B), confirming similar results published during the preparation of this paper (21, 22). Further, we found that the Δ*ipaH9*.*8* strain was deficient for cell-to-cell spread in parental HeLa but not in *GBP1*^KO^ cells (Fig. 8C), indicating that IpaH9.8-mediated interference with GBP1 targeting is critical for *S. flexneri* dissemination throughout the colonic epithelium. Cell-to-cell spread of the Δ*mxiE* mutant compared to that of wild-type *S. flexneri* was still moderately diminished in *GBP1*^KO^ cells, suggesting that one or more additional *mxiE*-controlled effectors other than *ipaH9.8* promote cellular dissemination (Fig. 8C).

## DISCUSSION

Many cell-autonomous defense mechanisms depend on the ability of the host cell to detect the precise location of a microbe inside an infected host cell (42). Pathogen-containing vacuoles are targeted by GBPs following immune recognition through missing-self or aberrant-self mechanisms (30, 43, 44), but the mechanism by which GBPs detect cytosolic pathogens had not previously been investigated. Here, we demonstrate that human GBP1 contains a unique triple-arginine motif within its flexible C-terminal region, which mediates the delivery of GBP1 to the Gram-negative cytosolic pathogen *S. flexneri*. We also demonstrate in this study that rough *S. flexneri* mutants lacking LPS O antigen are targeted less efficiently by GBP1 than the coisogenic wild-type strain. These observations suggest that the triple-arginine motif of human GBP1 either directly or indirectly interacts with the polysaccharide portion of LPS on the bacterial surface.

GBP1 binding to bacteria still occurs in the absence of O antigen, albeit less efficiently than in its presence. One possible explanation for this observation is that GBP1 associates with LPS through two or more distinct interactions, as has been described for galectin-3. Galectin-3 binds to LPS through a high-affinity interaction with O antigen as well as a low-affinity interaction with the inner LPS core (33). Alternatively, GBP1 might be recruited to bacteria through an additional LPS-independent mechanism. Future *in vitro* binding studies are required to test whether GBP1 binds to Gram-negative bacteria directly or indirectly and to identify the specific binding substrate(s) or interaction partner(s) of the C-terminal triple-arginine motif essential for immune targeting of cytosolic *S. flexneri* by GBP1.

Previous work demonstrated that deposition of murine GBP2 on the cytosolic bacterial pathogen *Francisella novicida* results in bacteriolysis (13, 14). Similarly, murine GBPs also promote the lytic destruction of *S. flexneri* (21). Unexpectedly, we found that the delivery of human GBP1 to cytosolic *S. flexneri* is insufficient to kill bacteria or halt their replication. Instead, we observed that GBP1-bound bacteria fail to form actin tails and are consequentially restricted for efficient cell-to-cell spread. These observations were confirmed by an independent report published during the preparation of our manuscript (22). The differences between mouse and human with regard to the consequences of GBP targeting are intriguing and warrant future investigations to determine, for instance, whether the lytic pathway present in mouse cells is absent from human cells. The latter model is supported by a report demonstrating that GBP-dependent recruitment of the IFN-inducible GTPase Irgb10 to cytosolic *F. novicida* is required for bacteriolysis in mouse cells (45). Importantly, genes encoding Irgb10 and its paralogous subset of immunity-related GTPases (IRGs) with a canonical GXXXXGKS P-loop sequence are absent from the human genome (46, 47), potentially accounting for the absence of an intracellular bacteriolytic pathway in human cells.

The dysregulation of cell-autonomous defense programs by *S. flexneri* is paramount to the microbe’s ability to colonize its human host and establish an infection (48). In agreement with two studies published during the preparation of our manuscript (21, 22), we found that the translocated bacterial ubiquitin E3 ligase IpaH9.8 inhibits the association of GBP1 with cytosolic *S. flexneri* in human cells. We did not investigate the mechanism by which IpaH9.8 interferes with GBP1 localization to bacteria, but the aforementioned studies demonstrated that IpaH9.8 ubiquitinates GBP1 and promotes its degradation via the proteasome (21, 22). While proteolytic degradation was clearly demonstrated for infections with a high multiplicity of infection (MOI) or in cells ectopically expressing IpaH9.8 (21, 22), we failed to observe a pronounced decrease in GBP1 protein expression in cells infected with *S. flexneri* under the infection conditions used in our studies (Fig. 7C). Furthermore, video microscopy data suggest that GBP1 ubiquitination by IpaH9.8 helps to dislodge GBP1 from targeted bacteria (22). We therefore propose that ubiquitination by IpaH9.8 not only tags GBP1 for proteolytic degradation but also directly blocks binding of GBP1 to bacteria and/or removes GBP1 from already-targeted bacteria. Importantly, Li et al. demonstrated that IpaH9.8 promotes polyubiquitination of the GBP1 residues K582 and K587 (21), which flank the triple-arginine motif essential for recruiting GBP1 to *S. flexneri*. Whether PBM ubiquitination blocks GBP1 binding to bacteria through steric hindrance will be investigated in future studies.

## MATERIALS AND METHODS

### Cell lines and culture.

Primary wild-type and *galectin-3*^KO^ (49) MEFs were made from C57BL/6J background mice acquired from The Jackson Laboratory. MEFs, A549, HEK 293T, and HeLa cells were maintained at 37°C and 5% CO_2_ in Dulbecco modified Eagle medium (DMEM) supplemented with 10% heat-inactivated fetal bovine serum (FBS), nonessential amino acids (Gibco), and 55 μM β-mercaptoethanol. *GBP1*^KO^ lines were derived from HeLa-Cas9 cells, which contain a stably integrated Cas9 expression vector, lentiCas9-Blast (50). HeLa-Cas9 cells were transiently transfected with two guide RNAs directed against exon 6, GBP1 to -2 (TTGATCGGCCCGTTCACCGC) and GBP1 to -3 (TCCGGATACAGAGTCTGGGC), to introduce a predicted 64-bp deletion. Single clones were isolated by dilution. Resulting alleles are described in Table S1 in the supplemental material. All instances of IFN-γ priming were done overnight at 200 U/ml.

10.1128/mBio.01979-17.3 TABLE S1  *GBP1* alleles in HeLa *GBP1*^KO^ clones 1 and 2. Download TABLE S1, DOCX file, 0.05 MB. Copyright © 2017 Piro et al. 2017 Piro et al. This content is distributed under the terms of the Creative Commons Attribution 4.0 International license.

### Bacterial strains and infections.

Bacterial strains are summarized in Table S2. 2457T *S. flexneri*-derived *ΔmxiE*, *ΔospB*, *ΔospC1*, *ΔvirA*, *ΔIpaH1.4*, *ΔIpaH4.5*, *ΔIpaH7.8*, and *ΔIpaH9.8* mutants were constructed using the λ red recombinase-mediated recombination system (51) with the DNA oligomers listed in Table S3. GFP^+^ *S. flexneri* stains contain the vector pGFPmut2 (52). The IPTG-inducible GFP vector pRK2-IPTG-GFP was a gift from Wendy Picking. *S. flexneri* was grown at 37°C on tryptic soy agar plates containing 0.01% Congo red, as well as 50 µg/ml carbenicillin for pGFPmut2- and PRK2-IPTG-GFP-containing strains. For infections, 5 ml tryptic soy broth (TSB) was inoculated with a single Congo red-positive colony and grown overnight at 37°C with shaking. Saturated cultures were diluted 1:50 in 5 ml fresh TSB and incubated for 2.5 to 3 h at 37°C with shaking. Bacteria were diluted in prewarmed cell culture medium and spun onto cells for 10 min at 700 × *g*. Plates were incubated at 37°C for 30 min and then washed twice with Hanks balanced salt solution (HBSS), followed by addition of cell culture medium containing 25 µg/ml gentamicin and further incubation at 37°C. Unless otherwise specified, cells were infected at an MOI of 50. For time-lapse microscopy and plaque assays, 5 × 10^7^ bacteria were harvested by centrifugation and incubated in 1 ml phosphate-buffered saline (PBS) containing 5 μg/ml poly-d-lysine at 37°C for 15 min with shaking, as described previously (53). Poly-d-lysine-pretreated bacteria were then used for infection, as described above. The *B. thailandensis* wild-type strain E264 carrying a GFP expression construct was a gift from Edward Miao. *B. thailandensis* plus GFP was grown at 37°C on LB plates containing 100 µg/ml trimethoprim. For infections, overnight cultures were diluted 1:20 in LB plus trimethoprim and grown for 3 h at 37°C with shaking. Cells were infected at an MOI of 100, as described above. Infected plates were incubated at 37°C for 2.5 h, washed twice with HBSS, and incubated in the presence of 30 μg/ml kanamycin for 2 h. Kanamycin-containing medium was then replaced with medium without antibiotics, and the plates were incubated at 37°C to 8 hpi. GFP^+^ wild-type *L. monocytogenes* strain 10403S was previously described (54); it was grown at 37°C on brain heart infusion (BHI) plates containing 10 µg/ml streptomycin and 7.5 µg/ml chloramphenicol. Infections were carried out in a manner identical to that used for *S. flexneri* by using saturated overnight cultures grown at 37°C with shaking. Cells were infected with *L. monocytogenes* at an MOI of 5.

10.1128/mBio.01979-17.4 TABLE S2  List of bacterial strains used in this study. Download TABLE S2, DOCX file, 0.1 MB. Copyright © 2017 Piro et al. 2017 Piro et al. This content is distributed under the terms of the Creative Commons Attribution 4.0 International license.

10.1128/mBio.01979-17.5 TABLE S3  List of DNA oligomers used to generate *S. flexneri* mutant strains. Download TABLE S3, DOCX file, 0.1 MB. Copyright © 2017 Piro et al. 2017 Piro et al. This content is distributed under the terms of the Creative Commons Attribution 4.0 International license.

### Design of GBP expression constructs.

Vectors containing mCherry-tagged constructs are derivatives of pmCherry-C1 (Clontech) and were constructed using the DNA oligomers and restriction endonucleases listed in Table S4. For the chimeric constructs GBP1/2^CTSD^ and GBP2/1^CTSD^, two synonymous mutations were introduced into GBP1 codons 478 and 479 of pmCherry-GBP1 using QuikChange site-directed mutagenesis with GBP1-BclI oligomers (Table S5). These mutations introduced a BclI restriction endonuclease site within the sequence encoding the flexible region between helices 11 and 12. Following propagation of pmCherry-GBP1^BclI^ and pmCherry-GBP2 in *dam-* and *dcm*-negative *E. coli* cells (New England Biolabs), BclI digestion and T4 ligase-dependent repair were used to exchange the fragment encoding the C-terminal regions of the two proteins. All other mCherry-tagged chimeric and mutant constructs were constructed using QuikChange site-directed mutagenesis with the primers listed in Table S5. GBP2^+PBM+GBP1-CaaX^ was constructed stepwise from pmCherry-GBP2^+PBM^ mutated with GBP2/1^CaaX^ and then GBP2^I587A^ oligomers. Tetracycline-inducible GBP1 was constructed by inserting the *GBP1* open reading frame into the third-generation Tet-on vector pInducer20 (55), using Gateway Cloning Technology with the GBP1-*attB* oligomers listed in Table S5. GBP1^R584–586A^ was constructed by mutating the entry vector pDONR221-GBP1 via QuikChange site-directed mutagenesis using GBP1^R584–586A^ oligomers prior to Gateway LR reactions.

10.1128/mBio.01979-17.6 TABLE S4  List of oligomers and restriction sites used to generate mCherry-GBP fusion expression constructs. Download TABLE S4, DOCX file, 0.1 MB. Copyright © 2017 Piro et al. 2017 Piro et al. This content is distributed under the terms of the Creative Commons Attribution 4.0 International license.

10.1128/mBio.01979-17.7 TABLE S5  Oligomers used to generate GBP mutant and chimeric variants. Download TABLE S5, DOCX file, 0.2 MB. Copyright © 2017 Piro et al. 2017 Piro et al. This content is distributed under the terms of the Creative Commons Attribution 4.0 International license.

### Immunofluorescence microscopy, actin tail analysis, time-lapse microscopy, and CPP assays.

For standard microscopy, cells were fixed in 4% paraformaldehyde and permeabilized with 0.25% Triton X-100 in PBS. Coverslips were probed with a 1:150 dilution of a rabbit monoclonal antibody against human GBP1 (Abcam, Inc.; ab131255) and/or a 1:50 dilution of a mouse monoclonal antibody against LPS (RayBiotech; DS-MB-01267) and then with Alexa Fluor-conjugated secondary antibodies and 4 µg/ml of the nuclear dye Hoechst, where appropriate. For actin tail analysis, 1:40 phalloidin conjugated to Alex Fluor 488 was added to the secondary antibody mix. For each field of view, Z-stacks were taken at 0.5-μm intervals on a Zeiss 710 inverted confocal microscope to encompass the thickness of the cells. The percentage of bacteria with tails was determined by referencing across the collected Z-stacks through a blind analysis. For time-lapse microscopy, *GBP1*^KO^ HeLa cells were plated in 35-mm glass-bottom plates (MatTek) and transfected with pmCherry-GBP1. Cells were infected with GFP^+^ *S. flexneri* pretreated with poly-d-lysine at an MOI of 10. Cells were imaged in DMEM without phenol red but with 5% FBS and 25 µg/ml gentamicin. Calcium phosphate precipitate (CPP) assays were carried out in HEK 293T cells stably expressing yellow fluorescent protein (YFP)–galectin-3 and transiently transfected with mCherry-tagged GBP constructs, as described in reference 30. CPP assays were carried out in HEK 293T cells stably expressing YFP–galectin-3 and transiently transfected with mCherry-tagged GBP constructs, as described in reference 30.

### IPTG-inducible GFP viability assay.

HeLa cells were stimulated overnight with 200 U/ml IFN-γ and infected with *S. flexneri* containing the IPTG-inducible GFP vector pRK2-IPTG-GFP. At 2 hpi, IPTG was added to a concentration of 1 mM. Chloramphenicol was also added to a concentration of 60 µg/ml for negative controls. Coverslips were incubated for an additional 2 h before being fixed and stained with anti-LPS and anti-GBP1 antibodies, as described above.

### *S. flexneri* cluster analysis.

Three-color Z-stack images were acquired on a Zeiss 710 inverted confocal microscope. For cluster analysis, these images were separated into individual color Z-stacks. Z-stacks not containing bacterial signal were discarded. The image was then examined for the threshold to remove background signals, and the three-dimensional object counter (3D-OC) plugin (ImageJ) (56) was used to identify connected objects. To exclude nonspecific signals, an object was defined as a minimum of 10 connected voxels above a set signal threshold and each object was assigned a unique tag. The average size of an individual bacterium was approximated to be 50 voxels. Objects with a volume of greater than 150 voxels (3 or more bacteria) were defined as a cluster.

### Plaque assays.

Plaque assays were performed essentially as previously described (57). Cells were plated to confluence in 35-mm glass-bottom plates and stimulated overnight with 200 U/ml IFN-γ. Cells were infected at an MOI of 5 × 10^−4^ with GFP^+^ *S. flexneri* pretreated with poly-d-lysine. Following 30 min of infection at 37°C, plates were washed twice with HBSS and overlaid with 1.5 ml DMEM without phenol red supplement but with 5% FBS containing 25 µg/ml gentamicin, 200 U/ml IFN-γ, 50 µg/ml carbenicillin, and 0.5% agarose. Plates were incubated for 10 min at room temperature before addition of 1 ml of cell culture medium without agarose. Plaques were visualized using phase-contrast and fluorescence microscopy at 24 hpi, and Feret’s diameter of individual bacterial plaques was determined using FIJI software. For plaque assays using *GBP1*^KO^ cells complemented with pInducer-GBP1 or -GBP1^R584−586^, cells were stimulated overnight with 1 µg/ml aTc.

### Titration of GBP1 expression from a Tet-inducible expression system and immunoblots.

*GBP1*^KO^ cells stably transduced with pInducer-GBP1 constructs were incubated overnight with cell culture medium containing the concentrations of aTc indicated in Fig. 6 and 7. Cells were lysed in radioimmunoprecipitation assay (RIPA) buffer containing 1× protease inhibitor cocktail (Sigma) for 5 min on ice. Lysates were spun for 10 min at maximum speed in a microcentrifuge at 4°C and then combined with an equivalent volume of 2× Laemmli sample buffer containing β-mercaptoethanol and incubated at 95°C for 10 min. Samples were run on 4 to 15% Mini-Protean TGX gels (Bio-Rad) and transferred to nitrocellulose. Immunoblots were probed with 1:1,000 anti-GBP1 (Abcam, Inc.; ab131255), 1:1,000 anti-mCherry rabbit polyclonal (Abcam, Inc.; ab183628), or anti-GAPDH (glyceraldehyde-3-phosphate dehydrogenase) rabbit polyclonal (Abcam, Inc.; ab9485) antibody, followed by horseradish peroxidase-conjugated anti-rabbit IgG (Invitrogen).

### Statistical analyses

Data analysis was performed using GraphPad Prism 6.0 software. Data shown are means ± standard errors of the means (SEM) unless otherwise indicated. Statistical significance was calculated using one-way or two-way analysis of variance (ANOVA) or Student’s *t* test, as indicated. Significance was defined as follows: ****, *P* < 0.0001; ***, *P* < 0.001; **, *P* < 0.01; and *, *P* < 0.05.
