# Supplementary figures and images for: Detection of Cytosolic Shigella flexneri via a C-Terminal Triple-Arginine Motif of GBP1 Inhibits Actin-Based Motility
Source: mBio. 2017 Dec 12;8(6):e01979-17. doi: 10.1128/mBio.01979-17 (PMC5727416; doi:10.1128/mBio.01979-17)

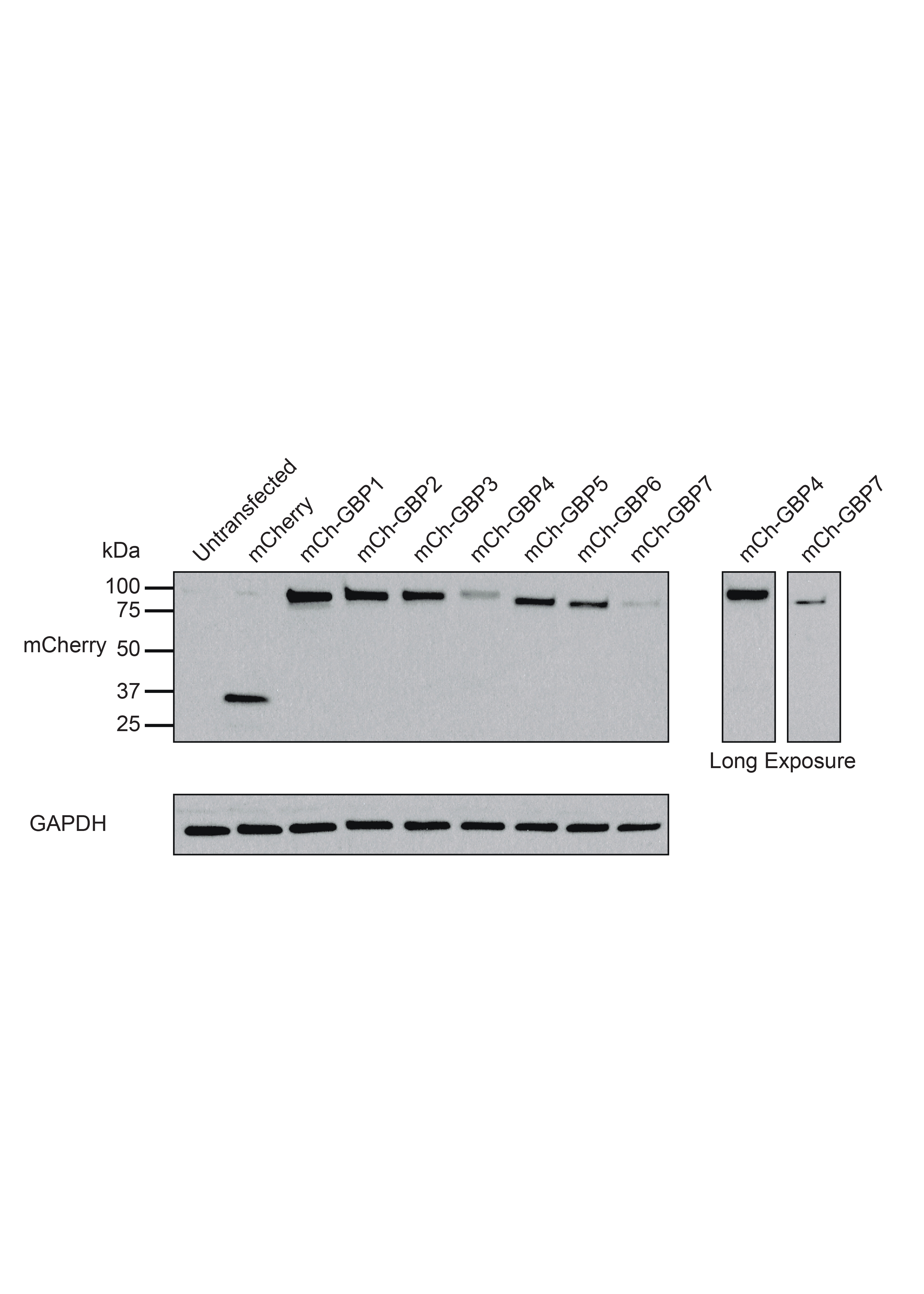

Supplement: FIG S1 [file mbo006173640sf1.jpg]

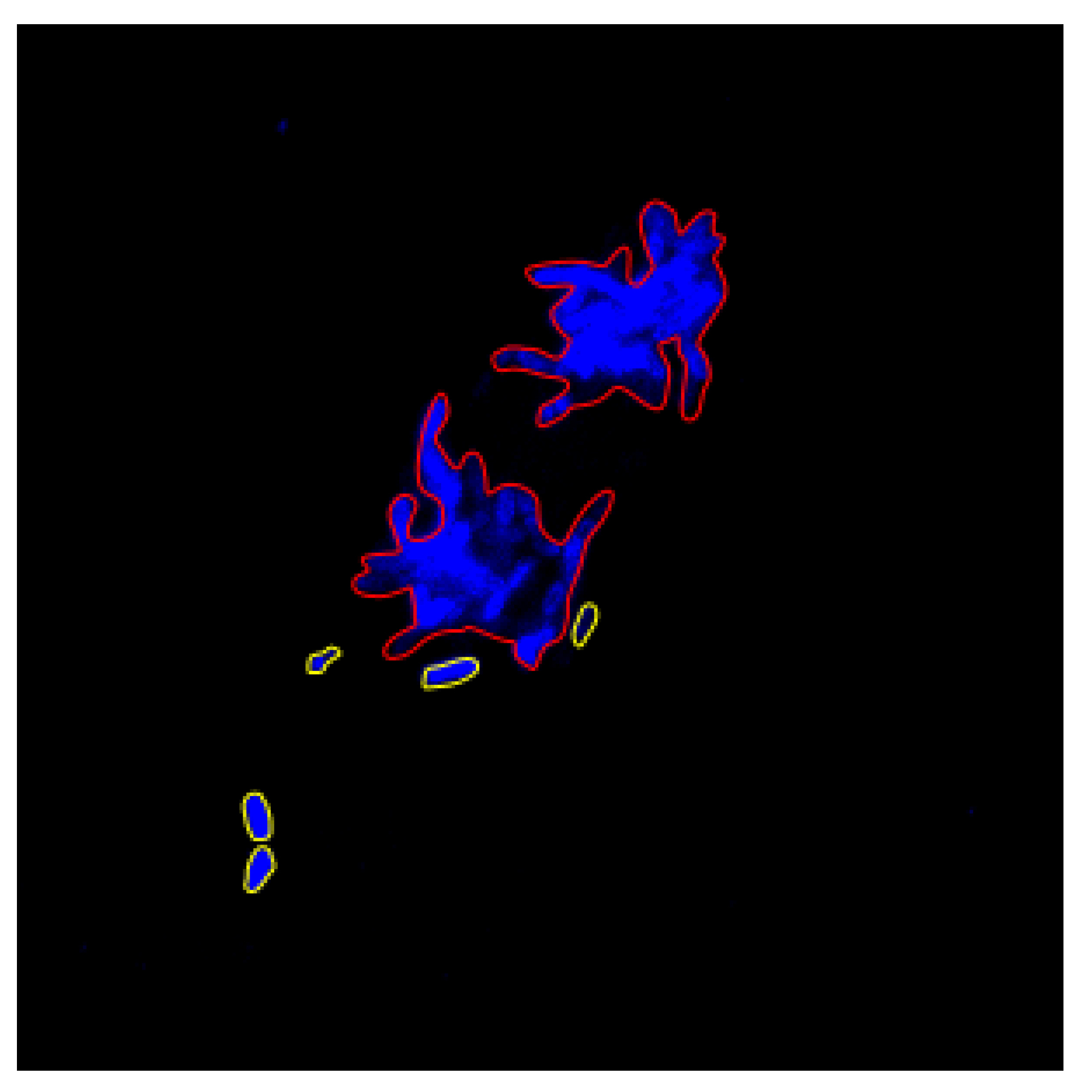

Supplement: FIG S2 [file mbo006173640sf2.jpg]
