# Supplementary material for: Detection of Cytosolic Shigella flexneri via a C-Terminal Triple-Arginine Motif of GBP1 Inhibits Actin-Based Motility
Source: mBio. 2017 Dec 12;8(6):e01979-17. doi: 10.1128/mBio.01979-17 (PMC5727416; doi:10.1128/mBio.01979-17)
Supplement: TABLE S1 [file mbo006173640st1.docx]

| **Mutation** | **Notes** | **Protein Size** |
| --- | --- | --- |
| deletion 668-729 | Deletion resulting in frame shift and early stop codon | 234 aa |
| deletion 667-730 | Deletion resulting in frame shift and early stop codon | 245 aa |

**Table S1. *GBP1* alleles in HeLa *GBP1*^KO^ clones #1 and #2.** Deletion is annotated based on the corresponding nucleotide positions in GBP1 ORF. The predicted protein size of truncated protein resulting from the mutation is provided (full-length GBP1 is 592 amino acids in length).
