# Supplementary material for: Detection of Cytosolic Shigella flexneri via a C-Terminal Triple-Arginine Motif of GBP1 Inhibits Actin-Based Motility
Source: mBio. 2017 Dec 12;8(6):e01979-17. doi: 10.1128/mBio.01979-17 (PMC5727416; doi:10.1128/mBio.01979-17)
Supplement: TABLE S2 [file mbo006173640st2.docx]

| **Species** | **Strain** | **Characteristics** | **Reference** |
| --- | --- | --- | --- |
| *S.flexneri* | WT | 2457T | [1] |
| *S.flexneri* | *ΔicsA* | 2457T *ΔicsA* | [2] |
| *S.flexneri* | *galU* | 2457T with mutant *galU* | [3] |
| *S.flexneri* | *rfaL* | 2457T with mutant *rfaL* | [3] |
| *S.flexneri* | *ΔmxiE* | 2457T *ΔmxiE* | This work |
| *S.flexneri* | *Δspa15* | 2457T *Δspa15* | [4] |
| *S.flexneri* | *ΔospB* | 2457T *ΔospB* | This work |
| *S.flexneri* | *ΔospC1* | 2457T *ΔospC1* | This work |
| *S.flexneri* | *ΔospE1/2* | 2457T *ΔospE1/2* | [5] |
| *S.flexneri* | *ΔospF* | 2457T *ΔospF* | [6] |
| *S.flexneri* | *ΔvirA* | 2457T *ΔvirA* | This work |
| *S.flexneri* | *ΔipaH1.4* | 2457T *ΔipaH1.4* | This work |
| *S.flexneri* | *ΔipaH4.5* | 2457T *ΔipaH4.5* | This work |
| *S.flexneri* | *ΔipaH7.8* | 2457T *ΔipaH7.8* | This work |
| *S.flexneri* | *ΔipaH9.8* | 2457T *ΔipaH9.8* | This work |
| *B.thailandensis* | WT | American Type Culture Collection 700388 expressing GFP | [7] |
| *L.monocytogenes* | WT | 10403S expressing GFP | [8] |

**Table S2. List of bacterial strains used in this study**
