# Supplementary material for: Detection of Cytosolic Shigella flexneri via a C-Terminal Triple-Arginine Motif of GBP1 Inhibits Actin-Based Motility
Source: mBio. 2017 Dec 12;8(6):e01979-17. doi: 10.1128/mBio.01979-17 (PMC5727416; doi:10.1128/mBio.01979-17)
Supplement: TABLE S3 [file mbo006173640st3.docx]

| **Oligomers** | **Sequence** (5’ to 3’) |
| --- | --- |
| MxiE-5' | CTATCATGAGAATGATCTTCGGGATAGTTGAATACCGAAGTGTAGGCTGGAGCTGCTTC |
| MxiE-3' | CTAACCGCCAAGTGTTCAGTTGTTCTTTGACTTTTGCCCCCATATGAATATCCTCCTTAG |
| OspB-5' | TTTATAAAACAATATATGGAGTCATGTAGGTATGAATTTAGTGTAGGCTGGAGCTGCTTC |
| OspB-3' | CCCTCCATAGCCTGATACAGGCTGTCCAGCTTTATGGGGTCATATGAATATCCTCCTTAG |
| OspC1-5' | ATTAAAACTGTTTTCATATAAGGTTCATTTTATGAATATAGTGTAGGCTGGAGCTGCTTC |
| OspC1-3' | CTGCCTTTTGCTAAACGATATTCAATTTTGATTAAATATACATATGAATATCCTCCTTAG |
| VirA-5' | ATTAATAGGAAAATACATCAGGAGAAATCAAATGCAGACAGTGTAGGCTGGAGCTGCTTC |
| VirA-3' | TTTACAGTCTGGCAGCCAATATAATATTGGCTTAAACATCCATATGAATATCCTCCTTAG |
| IpaH1.4-5' | ATCTGGTTAACCCATATACAAGGGAGACAGAATGACCGAAGTGTAGGCTGGAGCTGCTTC |
| IpaH1.4-3' | GGGGGGTGCCCCTATTAAGTTGTCAAGCATGTTATGACCCCATATGAATATCCTCCTTAG |
| IpaH4.5-5' | GACCAAGATATGAATAGTGAGGGGTTAATAAATGAAACCGGTGTAGGCTGGAGCTGCTTC |
| IpaH4.5-3' | GAAGTTTAGTCTCCAGGATTCCCGGGGCGGTTCAGGCCAGCATATGAATATCCTCCTTAG |
| IpaH7.8-5' | ATTCTCACAAATATAAGGTGGACCTAGCATTATGTTCTCTGTGTAGGCTGGAGCTGCTTC |
| IpaH7.8-3' | CCGGTCTGCGGTTTATGCTTATGCGACGTGATTATGAATGCATATGAATATCCTCCTTAG |
| IpaH9.8-5' | AACTCCTACTTATTTCTTTTAACAAAGCCATTTGTCCACCGTGTAGGCTGGAGCTGCTTC |
| IpaH9.8-3' | TCACTGGCGCTGACAGTTTTATGCGATGTGATTATGAATGCATATGAATATCCTCCTTAG |

**Table S3. List of DNA oligomers used to generate *S. flexneri* mutant strains**
