# Supplementary material for: Detection of Cytosolic Shigella flexneri via a C-Terminal Triple-Arginine Motif of GBP1 Inhibits Actin-Based Motility
Source: mBio. 2017 Dec 12;8(6):e01979-17. doi: 10.1128/mBio.01979-17 (PMC5727416; doi:10.1128/mBio.01979-17)
Supplement: TABLE S4 [file mbo006173640st4.docx]

| **Oligomer** | **Sequence** (5’ to 3’) | **Restriction Site** |
| --- | --- | --- |
| GBP1-F | AAGAACTAGATCTATGGCATCAGAGATCCACATGACAG | Bgl II |
| GBP1-R | CGGCCGCCCGGGTTAGCTTATGGTACATGCCTTTCGTCG | XmaI |
| GBP2-F | AAGAACTAGATCTATGGCTCCAGAGATCAACTTGCC | Bgl II |
| GBP2-R | CGGCCGCCCGGGTTAGAGTATGTTACATATTGGCTCCAATGATTTGC | XmaI |
| GBP3-F | CTGGAACTCGAGGGATGGCTCCAGAGATCCACATGAC | XhoI |
| GBP3-R | CGGCCGCCCGGGTTAGATCTTTAGCTTATGCGACATATATCTCTTGG | XmaI |
| GBP4-F | AAGAACTAGATCTATGGGTGAGAGAACTCTTCACGC | Bgl II |
| GBP4-R | CGGCCGCCCGGGTTAAATACGTGAGCCAAGATATTTTGTCCCT | XmaI |
| GBP5-F | CTGGAACTCGAGGGATGGCTTTAGAGATCCACATGTCAGA | XhoI |
| GBP5-R | CGGCCGCCCGGGTTAGAGTAAAACACATGGATCATCGTTATTAACAG | XmaI |
| GBP6-F | AAGAACTAGATCTATGGAATCTGGACCCAAAATGTTGG | Bgl II |
| GBP6-R | CGGCCGCCCGGGTTAAAAGGGGAGCTTATGCTTTTTAAAGAGTG | XmaI |
| GBP7-F | AAGAACTAGATCTATGGCATCAGAGATCCACATGC | Bgl II |
| GBP7-R | CGGCCGCCCGGGTCAGCTTATAATTTTCTTACCAGGATTTCTCAGC | XmaI |

**Table S4 List of oligomers and restriction sites used to generate mCherry GBP fusion expression constructs**
