# Supplementary material for: Detection of Cytosolic Shigella flexneri via a C-Terminal Triple-Arginine Motif of GBP1 Inhibits Actin-Based Motility
Source: mBio. 2017 Dec 12;8(6):e01979-17. doi: 10.1128/mBio.01979-17 (PMC5727416; doi:10.1128/mBio.01979-17)
Supplement: TABLE S5 [file mbo006173640st5.docx]

| **Oligomer** | **Sequence** (5’ to 3’) |
| --- | --- |
| GBP1-BclI-F | GACTGATGCAATTCTCCAGACTGATCAGACTCTCACAGAA |
| GBP1-BclI-R | TTCTGTGAGAGTCTGATCAGTCTGGAGAATTGCATCAGTC |
| GBP1^R48A^-F | GCAATTGTGGGCCTCTACGCCACAGGCAAATCCTACCTGATG |
| GBP1^R48A^-R | CATCAGGTAGGATTTGCCTGTGGCGTAGAGGCCCACAATTGC |
| GBP1^K51A^-F | ATTGTGGGCCTCTACCGCACAGGCGCATCCTACCTGATGAACAAG |
| GBP1^K51A^-R | CTTGTTCATCAGGTAGGATGCGCCTGTGCGGTAGAGGCCCACAAT |
| GBP1^S52N^-F | CTCTACCGCACAGGCAAAAACTACCTGATGAACAAGCTGGCT |
| GBP1^S52N^-R | AGCCAGCTTGTTCATCAGGTAGTTTTTGCCTGTGCGGTAGAG |
| GBP1^Δ589-592^-F | GAAAATGAGACGACGAAAGGCATAACCCGGGATCCA |
| GBP1^Δ589-592^-R | TGGATCCCGGGTTATGCCTTTCGTCGTCTCATTTTC |
| GBP1^C589A^-F | AGACGACGAAAGGCAGCCACCATAAGCTAACCCGGGAT |
| GBP1^C589A^-R | ATCCCGGGTTAGCTTATGGTGGCTGCCTTTCGTCGTCT |
| GBP1/2^CaaX^-F | CGAAAGGCATGTAACATACTCTAACCCGGGATCCACCGG |
| GBP1/2^CaaX^-R | CCGGTGGATCCCGGGTTAGAGTATGTTACATGCCTTTCG |
| GBP2/1^CaaX^-F | GAGCCAATATGTACCATAAGCTAACCCGGGATCCACCGG |
| GBP2/1^CaaX^-R | CCGGTGGATCCCGGGTTAGCTTATGGTACATATTGGCTC |
| GBP1^ΔPBM^-F | GATCTCCAGACGAAAGCATGTACCATAAGC |
| GBP1^ΔPBM^-R | GCTTATGGTACATGCTTTCGTCTGGAGATC |
| GBP1^R584-586A^-F | GAGATACAGGATCTCCAGACGAAAATGGCAGCAGCAAAGGCATGTACCATAAGCTAAC |
| GBP1^R584-586A^-R | GTTAGCTTATGGTACATGCCTTTGCTGCTGCCATTTTCGTCTGGAGATCCTGTATCTC |
| GBP2^+PBM^-F | GAGAAGCAAATCATTGGAGCCAAAAATGAGACGACGAAAGATATGTAACATACTC |
| GBP2^+PBM^-R | GAGTATGTTACATATCTTTCGTCGTCTCATTTTTGGCTCCAATGATTTGCTTCTC |
| GBP2^I587A^-F | AGAAGCAAATCATTGGAGCCAGCATGTAACATACTCTAACCC |
| GBP2^I587A^-R | GGGTTAGAGTATGTTACATGCTGGCTCCAATGATTTGCTTCT |
| GBP1-attB1-F | GGGGACAAGTTTGTACAAAAAAGCAGGCTGCCACCATGGCATCAGAGATCCACATGACAG |
| GBP1-attB2-R | GGGGACCACTTTGTACAAGAAAGCTGGGTCTTAGCTTATGGTACATGCCTTTCGTC |

**Table S5. Oligomers used to generate GBP mutant and chimeric variants.**
